# Comparison of Seven Healthy Lifestyle Scores Cardiometabolic Health: Age, Sex, and Lifestyle Interactions in the NutrIMDEA Web-Based Study

**DOI:** 10.1007/s44197-023-00140-1

**Published:** 2023-08-27

**Authors:** R. Ribot-Rodríguez, A. Higuera-Gómez, R. San-Cristobal, V. Micó, J. A. Martínez

**Affiliations:** 1 Precision Nutrition and Cardiometabolic Health, IMDEA-Food Institute (Madrid Institute for Advanced Studies), Campus of International Excellence (CEI) UAM+CSIC, 28049 Madrid, Spain; 2 Centre Nutrition, Santé et Société (NUTRISS), Institut sur la Nutrition et les Aliments Fonctionnels de l’Université Laval (INAF), Université Laval, Quebec, QC Canada; 3 School of Nutrition, Université Laval, Quebec, QC G1V 0A6 Canada; 4 CIBERobn Physiopathology of Obesity and Nutrition, Institute of Health Carlos III (ISCIII), 28029 Madrid, Spain

**Keywords:** Public health, Precision nutrition, Health-related quality of life, Physical health, Mental health

## Abstract

**Background:**

Global health status concerns both the assessment of wellbeing as well as the associated individualized determinants including quality of life and lifestyle factors. This study aimed to evaluate seven cardiometabolic health related scores and the influence, as well as interactions of lifestyle, heart-related and health-related quality of life (HRQoL) factors in order to inform the future implementation of precision public health (PPH).

**Methods:**

Data collected from 17,333 participants who were enrolled of the NutrIMDEA study. The data collection period was between May 2020 and November 2020 through an online survey. The baseline questionnaire collected information on socio-demographic data, cardiometabolic history, anthropometric variables and lifestyle aspects. Also, physical and mental component scores of SF12 Health Survey (PCS12/MCS12) were assessed as HRQoL features, which were applied to estimated seven scores (LS7, HLS, 20-years DRS %, FBS, CLI, WAI derived, LWB-I).

**Results:**

Most indices (except FBS, CLI, 20-years DRS % and WAI derived) showed that cardiometabolic outcomes and HRQoL measures were dependent on interactions by age and sex. The largest ponderal effect was found in PA total and Mediterranean Diet Score (MEDAS-14) interaction using LS7 as reference. However, using LWB-I as standard, the greatest effect was found in the quality-of-life feature MCS12. Noteworthy, LS7 showed good discrimination against PCS12, while LWB-I demonstrated excellent discrimination to MCS12.

**Conclusions:**

A major finding was the interplay between MEDAS-14 and PA on the LS7 scale as well as major effects of lifestyle factors and MCS12/PCS12 among scores, which need to be accounted with precision when implementing cardiometabolic screenings with PPH purposes.

## Introduction

Global health status involves the assessment of wellbeing and quality of life as well as the associated disease determinants [1, 2] including diet and physical activity (PA) [3, 4]. Identifying the level of dissimilarities between human groups regarding indicators is essential for featuring populations at risk and to promote policies [5]. Given the importance of quantifying lifestyle factors such as mediterranean diet adherence, PA, smoking habit, as well as non-modifiable factors such as age and sex, the use of healthy lifestyle scores and quality of life measures can be a suitable and useful tool for precision public health (PPH) population assessment [6, 7]. In this sense, several initiatives have been performed for the identification of different behavioral characteristics, such as health responsibilities, exercise, nutrition, stress management, promoting a healthy lifestyle [8, 9]. Consideration of endogenous and exogenous health components of a population allows the implementation of public health (PH) strategies through dietary recommendations and health promotion [3, 10]. In this context, the American Heart Association (AHA) published the population attributable fraction for cardiovascular disease (CVD) death. This study attributed a 40.6% to high blood pressure (HBP), 13.7% to smoking habit, 13.2% to inadequate diet, 11.9% to sedentary lifestyle and 8.8% to abnormal glucose rate [11]. On the other hand, the benefits of well-balance diet and the promotion of PA have been widely described [12, 13].

Actually, major risk factors as smoking habit, unhealthy diet, physical inactivity have an impact on metabolic dysfunctions, being a therapeutic target for the prevention of cardiometabolic related diseases such as hypertension, diabetes, dyslipidemia and obesity [14]. All mentioned factors have been integrated and categorized to implement a PPH in the target sample. Similarly, the healthy lifestyle scores, as a quantitative measure has been demonstrated an inverse association with CVD and reduction CVD risk more likely for young adults than for old adults [15], so it can be used as more efficient PH strategy.

Several instruments to measure health and quality of life have been developed to assess different objectives and characteristics of the population [16]. Self-administered or interviewer-administered questionnaires allow to evaluate differences between groups of patients [17]. Health measurements are especially important in the assessment of the consequences derived from chronic diseases, to monitor and identify the most relevant predictors in the disease development [16]. A commonly used approach to evaluate global health is the use of health-related quality of life (HRQoL) questionnaires, which included generic and specific instruments for certain populations or disorders [2]. In this regard, the Short-Form (SF-12) health questionnaire [18] is widely used to evaluate HRQoL. This form is based on the two main components of health, the physical scale (PCS12) and the mental scale (MCS12). The elements are computed in such a way that the higher score, the better the state of health. Additional instruments for quality of life estimations are EQ-5D-5L, Nottingham Health Profile or World Health Organization Quality of Life-BREF among others, while quantifying cardiometabolic health outcomes through health indicators, such as the Framingham score or Ideal Cardiovascular Index have been also used [19]. Additionally, online data collection initiatives, such as web-based or mobile applications, are increasing for PH management. These methods allow researchers to contact the population for the collection of information through questionnaires or the application of behavioral change therapies remotely. This allows to reach a larger population, reducing costs and time [20, 21].

This study aimed to evaluate seven multi-dimensional healthy-lifestyle scores designed to predict cardiometabolic health status as well as the interactions of lifestyle, heart-related and HRQoL factors in an online cohort-based study supported on validated questionnaires and scales to produce PH guidelines.

## Methods

### Participants and Procedures

This research was focused on comparing different healthy lifestyle scores and quality of life domains among recruited online adults. A cross-sectional study of 17,333 participants in NutrIMDEA Study 2020 [22] was conducted. The online recruitment period was between May 2020 and November 2020. Inclusion criteria considered participants with age over 18 years and acceptance of the survey completion.

The baseline questionnaire collected information on socio-demographic data, cardiometabolic history, anthropometric variables and lifestyle aspects. The survey was based on validated questionnaires [18, 23–25]. The questionnaire was freely online accessible at https://nutrimdea2020.questionpro.com/. Further characteristics of the sample and ethics considerations were described in previous article of NutrIMDEA 2020 Study [22]. The questionnaire was presented to IMDEA-CEI and the external companies that performed the surveys, which confirmed that participation in the questionnaire is a proof of acceptance to participate to the NutrIMDEA study with own anonymized data.

### Data Analyses and Self-Referred Questionnaires

Among sociodemographic data, the age was analyzed within two categories (< 40 years and > 40 years). Sex consisted of two categories (male and female). Ethnicity, educational level, job status and marital status were included in the analyses as independent variable or covariate as appropriate. Concerning anthropometric data, BMI was calculated using self-declared weight and height. Cardiometabolic disease prevalence and family history were self-reported by participants with the following options: diabetes, high blood pressure (HBP), dyslipidemia and obesity. Nutritional quality included the Mediterranean Adherence Diet Score by using the PREDIMED questionnaire as (MEDAS-14) [23]. Other lifestyle factors were collected as smoking habit and sleeping habit. PA was assessed using the International Physical Activity Questionnaire (IPAQ) short version in Spanish [26]. Quality of Life features were assessed with the SF12 and physical and mental component scores of SF12 were computed following the standard protocol [27].

### Healthy Lifestyle Scores Assessment

Life Simple 7 score (LS7) was defined as a combination of four modifiable behaviors: normal body mass index (BMI), adequate PA, high MEDAS-14, not smoking habit and three cardiometabolic factors: dyslipemia, HBP and diabetes [28]. Classification index was poor (0–3), intermediate (4) and excellent (5–7). Healthy Lifestyle Score (HLS) included five healthy habits: never having smoked, moderate to high PA, high MEDAS-14, moderate alcohol consumption and normal BMI [29]. This score was classified poor (0–1), intermediate (2) and excellent (3–5). 20-year cardiovascular disease risk score (20y-DRS) % predicts the 20-year percentage of cardiovascular risk separately for men and women with different ponderated equation. This score was based on lifestyle factors (BMI status, smoking habit, healthy diet score, PA, moderate alcohol intake) as described elsewhere [30, 31]. Fuster-BEWAT score (FBS) [32, 33] was computed using five indicators the prevalence of HBP, PA level, vegetables/fruits consumption, BMI, and smoking habit. This index was classified in three groups: poor (0–1), intermediate (2–3) and excellent (4–5). Composite Lifestyle Index (CLI) was developed by measuring six lifestyle components (PA, smoking habit, healthy diet score, moderate alcohol intake, adequate sleep and stress index) [34]. It was classified in three groups: low (0–20), intermediate (20–40) and high (40–60). Work Ability Index (WAI) derived was constructed based on WAI [35].This index included educational level, marital status, BMI status, smoking habit, healthy diet score, vegetables/fruit consumption, red meat consumption, PA and HRQoL. Lifestyle and Wellbeing Index (LWB-I) included sociodemographic, lifestyle and nutritional items: sex, age, BMI, PA, marital status, smoking habit, family history of diseases, red meat consumption, added sugars consumption, adequate sleep, insomnia, stress, HRQoL, vegetable/fruit consumption, healthy diet score and cardiometabolic diseases (dyslipemia, HBP, diabetes) [36]. This index was classified as poor (< 75 points), transitioning (75–81 points) and excellent (> 81 points). Features of all scores are summarized in Table 1.

**Table 1:**
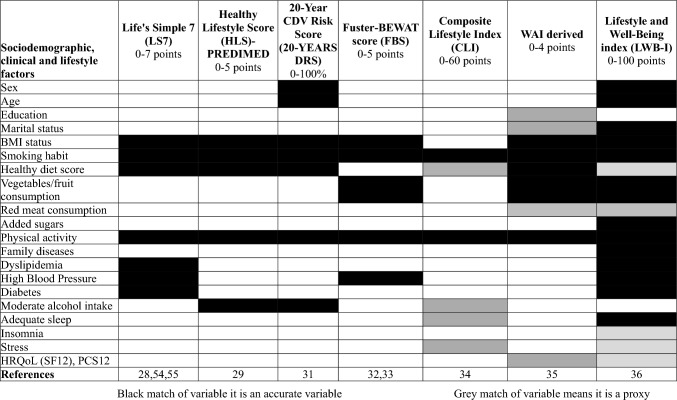
Comparison of variables that are measured on different lifestyle scores concerning cardiometabolic health

### Statistical Analyses

Statistical analyses were carried out using SPSS Statistics version 26 (SPSS Inc., Chicago, IL, USA). Comparisons of sociodemographic data, lifestyle and individual characteristics across categories of healthy lifestyle scores were performed using Students t test, or analysis of variance (ANOVA) with Tukey's post hoc for multiple comparisons concerning continuous variables. To evaluate the impact of the dietary and lifestyle habits on the HRQoL, a stratification of the sample was carried out as low/high MEDAS-14 and low/high PA, which were classified according the median. The reason of this approach involving these lifestyle factors was due to the fact that no threshold criteria has been set up for these variables, while the cut-off of age (40 years old) was based in common assumptions of this kind of surveys with a good distribution in our distribution (more than 6500 participants in each group). Statistically analysis for categorical variables was done using proportions analysis, using *χ*^2^ distribution.

A bivariate analyses were performed to identify candidate variables using a significance level of *p* < 0.05. Some explanatory variables with no significance were excluded (*p* > 0.05). The variables with *p* values < 0.05 for the Pearson correlation were entered into a series of multivariable models. Before fitting multivariable models for LS7, was examined multicollinearity among the explanatory lifestyle variables with the use of the variance inflation factor and Pearson correlation coefficients to ensure the absence of collinearity between the included variables. Sensitivity analyses have been performed as appropriate.

Logistic regression model with receiver operating characteristic (ROC) curve was fitted using PCS12 and MCS12 as gold standard versus all calculated scores were compared to assess the specificity and sensitivity using the area under the curve (AUC).

For the multiple linear regressions, a linear relationship between LS7/LWB-I and each continuous variable was analysed. Predictive variables tested by stepwise method were: age, sex, ethnicity, education, job status, smoking status, PA Total, MEDAS-14, PCS12, MCS12, self-reported health status, assuming that all variables were quantitative or categorical and the outcome variable was quantitative, continuous and unbounded. Differences were considered to be significant when *p* < 0.05. *β*-coefficients reflect the amount of LS7 points that are attributed to each individual variable. LS7 and LWB-I as dependent variables were used.

## Results

Participants were mainly over 40 years (61%), females (62.7%), have a higher educational level (83.9% more than High School), mainly workers (73.4%) and 58.3% presented normal weight. Cardiometabolic diseases prevalence in the sample was 17.1% obesity, 3.8% diabetes, 9.7% HBP, and 15.1% dyslipidemia. 19.1% are former smokers and 18.4% current smokers. Self-reported HRQoL showed a 16.8% state a poor/fair status, 56.5% good and 26.6% very good/excellent HRQoL. Meanwhile, the summarizing PCS12 mean was 53.51 points and the MCS12 mean was 43.86 points.

The different scores are reported (Table 2) according to age group (< 40 years and > 40 years) and sex (male and female). According to the age group, all scores had significative differences (except FBS) and the > 40 years group showed worse score than < 40 years. However, concerning sex classification, all scores showed significative differences and mainly males showed better scores than females, save LS7, CLI and WAI derived where scores in females were higher than in males. In addition, most scores fitted a significant interaction between age group and sex except CLI, WAI derived and FBS. Significant interactions were found in scores that include the variables BMI, smoking habit, healthy diet score and PA. According to HRQoL measures, PCS12 and MCS12 were included in Table 2. Participants < 40 years showed higher PCS12 [(54.6 (6.4) points)] than participants > 40 years [(52.9 (7.1)] with significative difference. However, participants > 40 years showed higher MCS12 [45.3 (10.1)] points. No significative differences in PCS12 between sex was found (*p* = 0.43). Interaction between age group (*p* < 0.001) and sex (*p* < 0.001) was detected in both PCS12 and MCS12.

**Table 2:** Different lifestyle scores and HRQoL measures of participants stratified by age group and sex

	Overall	≤ 40 years	> 40 years	*p* test age	Male	Female	*p* test sex	*p* test interaction
*n* (%)		6778 (39.1)	10,554 (60.9)		6403 (37.0)	10,862 (62.7)		
LS7 [mean (SD)]	4.48 (1.1)	4.68 (1.0)	4.34 (1.2)	** < 0.001**	4.24 (1.2)	4.65 (1.04)	** < 0.001**	**0.013**
LS7 qualitative classification [*n *(%)]				** < 0.001**			**0.035**	
0–3 (Poor)	2038 (18.6)	793 (17.0)	1245 (19.8)		827 (18.8)	1200 (18.5)		
4 (Intermediate)	4745 (43.4)	2167 (46.5)	2578 (41.1)		1850 (42.0)	2880 (44.4)		
5–7 (Ideal)	4162 (38.0)	1705 (36.5)	2457 (39.1)		1730 (39.3)	2410 (37.1)		
HLS [mean (SD)]	2.37 (0.8)	2.45 (0.87)	2.27 (0.8)	** < 0.001**	2.43 (0.9)	2.33 (0.8)	** < 0.001**	** < 0.001**
HLS-qualitative classification [*n* (%)]				** < 0.001**			** < 0.001**	
0–1 (Poor)	1544 (13.6)	717 (15.0)	827 (12.6)		584 (12.6)	953 (14.3)		
2 (Intermediate)	4816 (42.5)	2229 (46.7)	2587 (39.4)		1866 (40.4)	2931 (43.9)		
3–5 (Ideal)	4979 (43.9)	1831 (38.3)	3148 (48.0)		2171 (47.0)	2786 (41.8)		
CLI [mean (SD)]	36.91 (6.2)	37.58(5.8)	36.52 (6.3)	** < 0.001**	35.77 (6.4)	37.63 (5.9)	** < 0.001**	0.995
CLI qualitative classification [*n* (%)]				** < 0.001**				
0–20 (Poor)	71 ( 0.7)	13 ( 0.4)	58 (1.0)		35 (0.9)	36 ( 0.6)		
20–40 (Intermediate)	6339 (65.9)	2235 (63.0)	4104 (67.6)		2681 (71.9)	3635 (62.1)		
40–60 (Ideal)	3213 (33.4)	1301 (36.7)	1912 (31.5)		1012 (27.1)	2183 (37.3)		
FUSTER-BEWAT [mean (SD)]	2.10 (0.8)	2.10 (0.8)	2.09 (0.8)	0.380	2.14 (0.8)	2.06 (0.8)	** < 0.001**	0.552
FUSTER-BEWAT qualitative classification [*n* (%)]				** < 0.001**			** < 0.001**	
0–1 (Poor)	2308 (20.5)	875(18.4)	1433 (22.0)		888 (19.4)	1412 (21.2)		
2–3 (Intermediate)	8574 (76.1)	3744(78.6)	4830 (74.3)		3495 (76.4)	5043 (75.9)		
4–5 (Ideal)	387 ( 3.4)	145 ( 3.0)	242 ( 3.7)		193 (4.2)	190 ( 2.9)		
20 years DRS male (mean (SD))	4.44 (6.79)	0.18 (0.1)	2.00 (1.6)	** < 0.001**				
20 years DRS female [mean (SD)]	4.39 (11.2)	0.47 (1.1)	7.72 (14.3)	** < 0.001**				
WAI derived [mean (SD)]	2.33 (0.9)	2.64 (0.9)	2.21 (0.9)	** < 0.001**	2.10 (0.9)	2.50 (0.9)	** < 0.001**	0.356
LWB-I [mean (SD)]	73.78 (9.8)	74.95(9.7)	71.77 (9.8)	** < 0.001**	75.91 (9.5)	72.42 (9.8)	** < 0.001**	**0.01**
LWB-I qualitative classification [*n* (%)]				** < 0.001**				
< 75 points (Poor)	5003 (52.2)	2121 (59.9)	2882 (47.6)		1600 (43.0)	3382(58.0)		
75–81 points (Transitioning)	2358 (24.6)	870 (24.6)	1488 (24.6)		1013 (27.2)	1339(23.0)		
> 81 points (Excellent)	2228 (23.2)	548 (15.5)	1680 (27.8)		1107 (29.8)	1110(19.0)		
PCS12 (mean (SD))	53.5 (6.9)	54.6 (6.4)	52.9 (7.1)	** < 0.001**	53.5 (6.5)	53.5 (7.1)	0.430	** < 0.001**
MCS12 (mean (SD))	43.9 (10.7)	41.1 (11.2)	45.3 (10.1)	** < 0.001**	43.9 (10.7)	42.8 (10.9)	** < 0.001**	** < 0.001**

Data are mean (standard error) or percentage of participants. *P* value for comparisons between groups calculated with Chi-square tests for categorical variables or independent *t*-test for quantitative variables.﻿ Bold for *p*-values <0.05

Participants mainly showed low MEDAS-14 (66.4%), significative differences between low/high MEDAS-14 (*p* < 0.001) were found in all scores (except in 20-years DRS and CLI) and higher scores in participants with high MEDAS-14 (Table 3). There were significative differences between low/high PA in all scores (*p* < 0.001) except in 20-years DRS females *(p* = 0.17) and males (*p* = 0.56) and in WAI derived (*p* = 0.79). Also, higher scores were detected in participants with high PA. Regarding HRQoL measures, higher scores of PCS12 and MCS12 were located in high MDS group.

**Table 3:** Comparison of Lifestyle Scores and HRQoL measures stratified by low/high MEDAS-14 and low/high PA

	Low MEDAS-14	High MEDAS-14	*p* test MEDAS-14	Low PA	High PA	*p* test PA
*n* (%)	11,530 (66.5)	5803 (33.5)		8621 (49.8)	8702 (50.2)	
LS7 [mean (SD)]	3.80 (0.85)	4.72 (0.80)	** < 0.001**	4.08 (0.93)	4.29 (0.98)	** < 0.001**
HLS [mean (SD)]	2.03 (0.71)	2.91 (0.71)	** < 0.001**	2.28 (0.80)	2.48 (0.87)	** < 0.001**
FBS [mean (SD)]	2.06 (0.78)	2.11 (0.81)	** < 0.001**	2.03 (0.75)	2.18 (0.85)	** < 0.001**
20 years DRS%						
Females	4.92(12.1)	2.67 (7.21)	0.411	4.49 (11.5)	4.27 (10.8)	0.164
Males	4.77 (6.86)	3.78 (6.60)	0.284	4.29 (6.28)	4.63 (7.38)	0.556
CLI [mean (SD)]	35.07 (5.98)	37.51 (6.13)	0.258	35.93 (5.9)	38.28 (6.3)	** < 0.001**
WAI derived [mean (SD)]	2.03 (0.96)	2.43 (0.90)	** < 0.001**	2.36 (0.93)	2.43 (0.90)	0.079
LWB-I [mean (SD)]	71.36 (10.24)	74.56 (9.54)	** < 0.001**	72.94 (9.71)	75.53 (9.28)	** < 0.001**
PCS12 [mean (SD)]	52.99 (7.23)	54.43 (6.24)	** < 0.001**	54.10 (6.16)	54.93 (5.57)	** < 0.001**
MCS12 [mean (SD)]	43.01 (10.89)	45.32 (10.20)	** < 0.001**	43.92 (10.29)	45.26 (10.04)	** < 0.001**

High/low MEDAS-14 and high/low PA were established with the median. *P* values for two way analysis of variance (ANOVA) with Turkey’s post-hoc test between groups. Bold for *p*-values <0.05

**Fig. 1:**
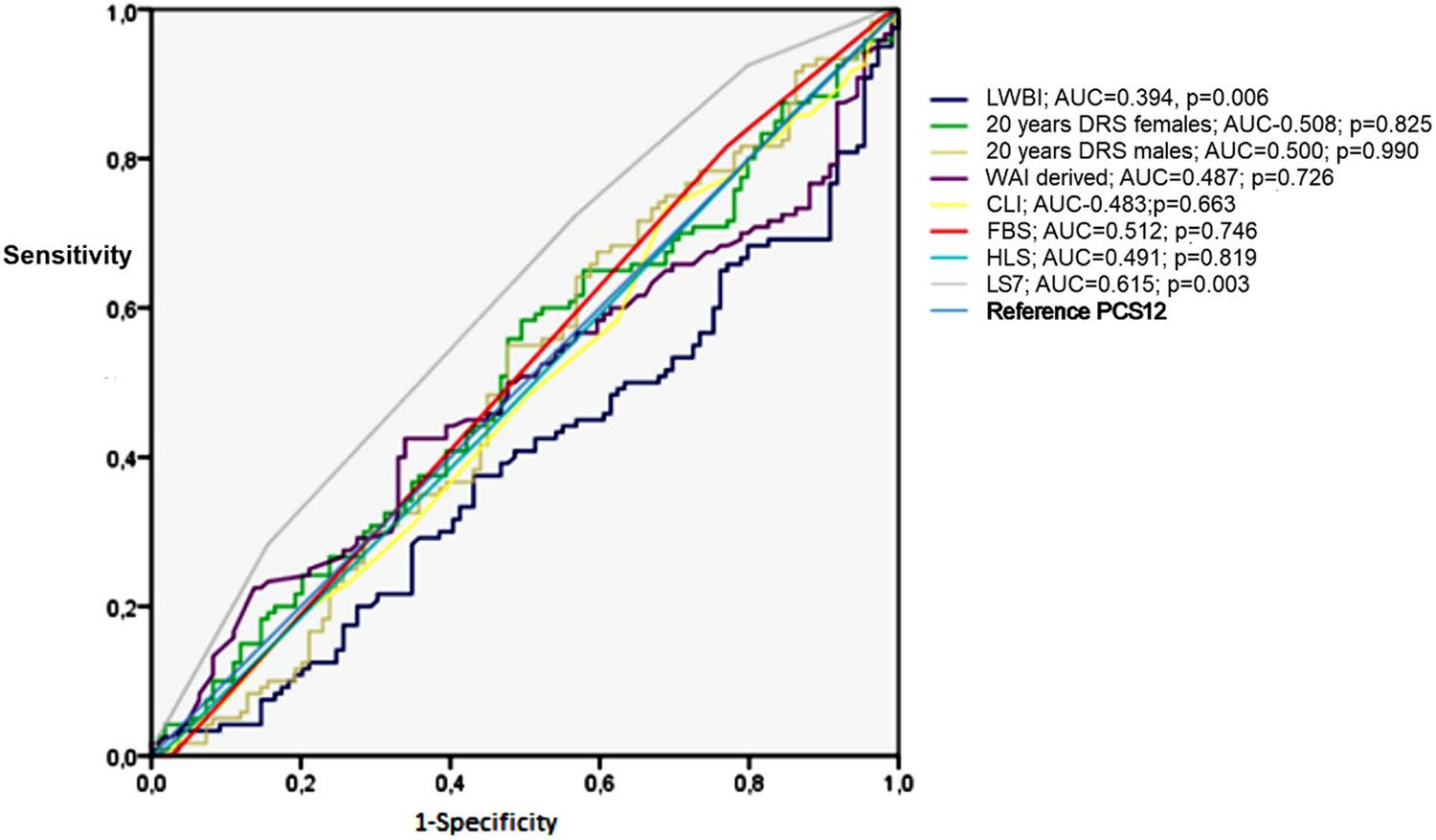
Logistic regression model Receiver operating characteristic (ROC) curve using PCS12 as reference. LS7 area under the curve (AUC) = 0.62; error 0.04; *p* = 0.003; CI 95% (0.54–0.69)

Attending to ROC analysis (Fig. 1), LS7 raised a significative discrimination for PCS12 (AUC = 0.63; *p* = 0.003). LWB-I revealed a significant discrimination (AUC = 0.84; *p* < 0.0001) for MCS12, while the others scores did not evidenced significative test.

**Fig. 2:**
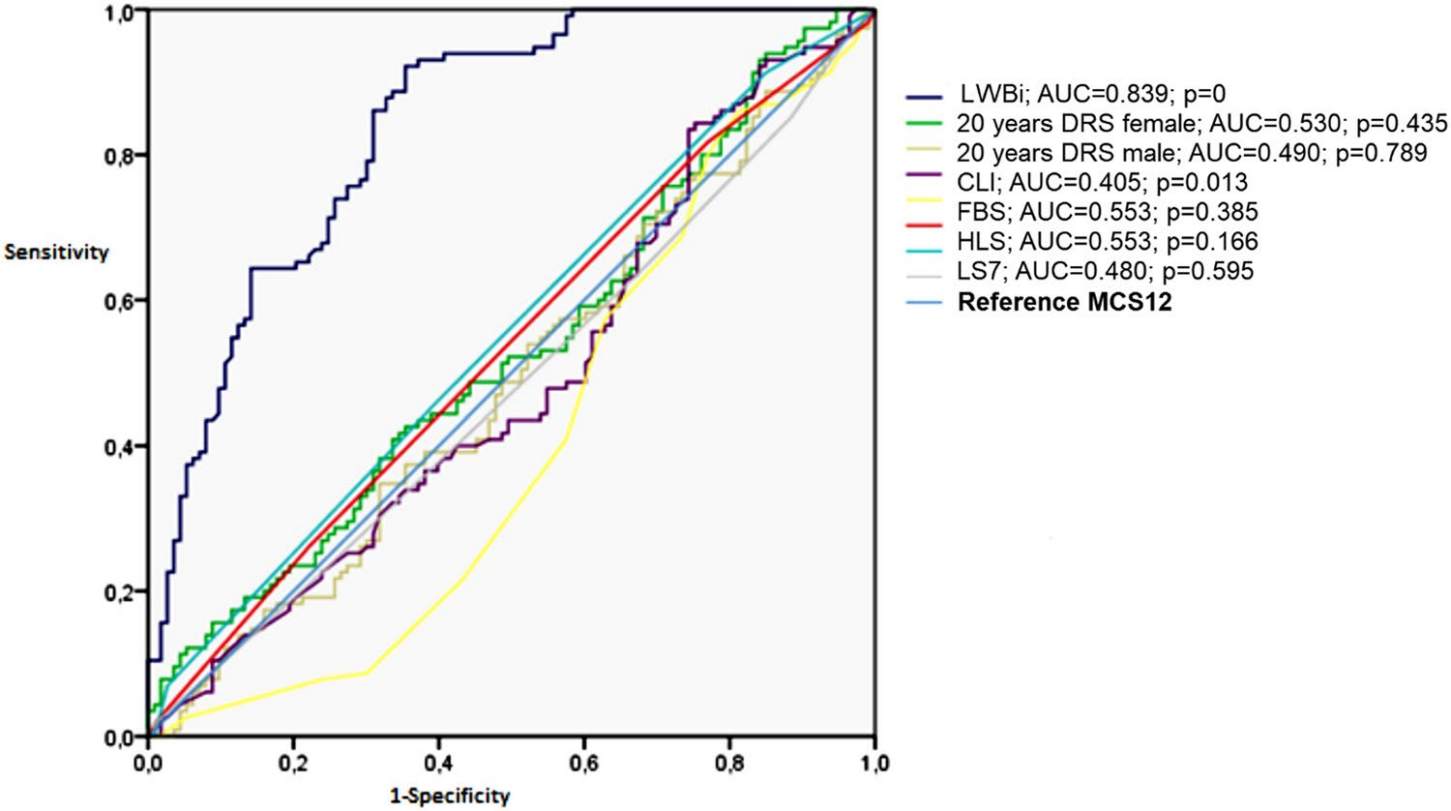
Logistic regression model receiver operating characteristic (ROC) curve using MCS12 as reference. LWB-I area under the curve (AUC) = 0.84; error = 0.026; *p* = 0; CI 95% (0.788–0.890)

To determine sociodemographic and lifestyle factors most influence in LS7 and LWB-I, linear regression analyses were performed. For LS7 (Table 4) a positive association with total PA and MDS interaction (*β* = 0.29), no smoker (*β* = 0.27), PCS12 (*β* = 0.12); a negative association with age (*β* = −0.19), male sex (*β* = −0.16) and inactive worker status (*β* = −0.03). Analysis of LWB-I (Table 5) showed a positive relationship with MCS12 (*β* = 0.69), PCS12 (*β* = 0.19), PA total and MDS interaction (*β* = 0.09) (Fig. 2).

**Table 4:** Multiple linear regression—predictor variables of LS7

Independent variables		
Sociodemographic variables		Standardized beta coefficient
Age (more than 40 years)		−0.19
Sex (Male)		−0.16
Education (less than High School)		−0.04
Job status (no worker)		−0.03
Lifestyle variables		
Smoking status (no smoker)		0.27
PA dichotomic (low PA)		−0.07
MEDAS-14 dichotomic (high MEDAS-14)		0.12
PA total and MEDAS-14 interaction		0.29
Quality of life features		
MCS12		0.04
PCS12		0.12
HRQoL (poor/fair self reported health)		−0.09

Dependent variable LS7. Constant = 2.91; *R*^2^ adjusted = 0.305, *R* = 0.552. *F* = 311.877. Significance *F* < 0.001. *p* value = 0 for all independent variables. The variables included in the model predict the dependent variable by 30.5%. Each *β*-coefficient translates to the expected LS7 points gained or lost per unit of sociodemographic variables (age, sex, education, job status), lifestyle variables (smoking status, PA total in hours/week, MEDAS-14) and quality of life features (MCS12, PCS12, poor self reported health and excellent self reported health). Ethnicity has been excluded of the model (*p* = 0.076)

**Table 5:** Multiple linear regression—predictor variables of LWB-I

Independent variables		
Sociodemographic variables		Standardized beta coefficient
Sex (male)		0.08
Ethnicity (Caucasian)		0.02
Job status (no worker)		0.03
Lifestyle variables		
MEDAS-14 dichotomic (high MEDAS-14)		0.03
PA total and MEDAS-14 interaction		0.09
Quality of life features		
MCS12		0.69
PCS12		0.17

Dependent variable LWB-I. Constant = 23.67. *R*^2^ adjusted = 0.502. *R* = 0.708. *F* = 1117.94. Significance *F* < 0.001. *p* value = 0 for all independent variables. The variables included in the model predict the dependent variable by 50.2%. Each *β*-coefficient translates to the expected LWB-I points gained or lost per unit of sociodemographic variables (age, sex, ethnicity, education, job status), lifestyle variables (smoking status, PA total in hours/week, MEDAS-14) and quality of life features (MCS12, PCS12, poor self reported health and excellent self reported health). Variables not included in table were excluded of the model. No significative variables: age (*p* = 0.06), education level (*p* = 0.18), Smoking habit (*p* = 0.18), PA (*p* = 0.28), HRQoL (*p* = 0.37)

## Discussion

Primary prevention of cardiometabolic diseases is an important strategy to reduce the incidence of cardiovascular events and premature death. Focus in the population awareness about health and the promotion of healthy lifestyles, environmental, social and economic determinants may reduce illness rates [37, 38]. To implement such objectives, it is necessary to develop tools that allow to identify populations and individuals exposed at risk for chronic diseases [39]. The main purpose of this research was to examine seven continuous scores and scales that had been proposed for estimating cardiometabolic health status and their determinants[40–44]. Health and diseased measurements for the evaluation of HRQoL and its changes throughout the life have been widely used in PH [45–48]. Thus, health indicators must have the power of discrimination, relevance, validity, sensitivity, specificity, reproducibility and interpretation [45]. Indeed, quantifying health allows precision medicine and the development of PPH policies [49]. In this context, a previous comparison of four healthy lifestyles scores demonstrated to be able to predict cardiovascular events related to PA and dietary habits [15].

As a measure of cardiometabolic health, each scale is different with own inherent features and limitations [16]. Available general health surveys allow an appraisal of the personalized nutritional status [50], although the best manner to implement this approach is to adequately combine epidemiological data. However, in PPH is a priority the use of validated health self-referred questionnaires to collect a large amount of information from the target population [51]. Thus, AHA recommends the LS7 questionnaire to characterize the cardiometabolic status due to a wide background scope and validated literature [11, 13, 52, 53]. Nevertheless, the other methodologies such as those described in this study are validated and characterized by different variables related to cardiometabolic health.

In this context, a large study performed in Europe found that participants with chronic diseases (CVD, cancer, respiratory disease, and diabetes) who had at least two risk factors (smoking, physical inactivity, and obesity) had a shorter life expectancy of about six years compared to those with none of these three risk factors [54]. In the SUN cohort, the combination of never smoking habit, high level of PA, high adherence to MedDiet, low BMI, moderate alcohol intake, and avoidance of binge drinking was associated with almost a 50% relative reduction in hypertension risk [18]. Furthermore, the PURE study [29] showed that the use of HRQoL questionnaires may prevent the blood testing to assess cardiovascular risk. This may be an advantage in regions with limited health resources, where the burden of CVD is alarmingly growing [55]. Additionally, the study of LS7 indicates that individuals with higher score on all seven metrics had a lower risk of CVD [19]. Similarly, it has been also found that improvement in these indices over time was associated with a lower risk of CVD in the future [56].

Our results support that increasing healthy lifestyle indices are related to higher levels of MEDAS-14 and PA, among other factors. Taking PCS12 as a reference, LS7 shows an acceptable discrimination. This finding is not surprising since this health indicator has shown a prevalence of good HRQoL increased linearly across high LS7 metrics [57]. Whereas, LWB-I, showed excellent discrimination against MCS12, by affording several questions of HRQoL [36]. However, the LS7 contains fewer questions (seven versus sixteen), which facilitates its application as nutritional screening and quick PPH implementation.

Interestingly, CLI was specifically able to detect PA and diet interactions, which may be associated to the behavioral issues requested in this questionnaire. In any case, LS7 and LWB-I scales accounted significantly sociodemographic, lifestyles and quality of life variables.

According to multivariate linear regression, non-smoking habit, high MEDAS-14, MCS12, PA total and female sex, respectively, have a greater influence on high score of LS7. Also is important to emphasize that in most studies [58–61], the MCS12 variable has not been evaluated and that it is ponderally relevant and valuable third variable with greater weight. The most influential variables are modifiable factors which confirm the impact of lifestyle features in the HRQoL level measured by validated questionnaires [2]. Moreover, healthy lifestyle scores are significantly correlated with CVD risk of death and they are considered as an interesting tool in health promotion [15, 56, 62] Similarly, some authors have suggested that the combination of different lifestyle factors may improve the predictor accuracy of the risk of non-communicable diseases^1^.

Recent initiative including online observational and intervention studies are becoming valuable in epidemiological field, such as Food4Me [21], SUN [63], Health Professionals and Nurses cohorts [64]. However, this type of study require some specific skills and the design of validated questionnaire to collect relevant health traits from the interviewed population [22, 65]. Interestingly, a previous online research concerning carbohydrates intake and PA revealed that glycemia depends on a conjoint impact of both nutritional and exercise determinants [66]. Indeed, a major finding of this research is the presence of interactions between PA and dietary intake involving LS7 and LWB-I outcomes. The two lifestyle variables cannot be considered independent, but one influences the other, and must be analyzed together. These results support that an online method could be a useful instrument to implement both population recommendations and individual advice concerning LS7 [28] and LWB-I scores [36, 42, 66]. Indeed, combined indication of healthy lifestyle factors could be more favorable in health outcomes [56]. This has been reported concerning the adherence to health determinants, including PA and behavioral risk factors [67] and sociodemographic status interactions [5], in a prospective cohort concerning all cause of death [1].

The present study has some strengths that must be mentioned. First, validated scores have been used [28, 29, 31, 32, 34–36], widely employed that can facilitate future comparisons. Also, a relatively large sample size has been included in the analyses. These tools can be applied in primary health care system and education as a tool to estimate the risk individuals to strategy in health promotion. However, the present study has some limitations as causality in the observed associations cannot be ascertained due to be an observational study with a non-representative sample as well as the online recruitment could suppose a technological bias in some population groups.

## Conclusions

Findings from this study suggest certain differences between healthy lifestyle scores concerning cardiometabolic health and comparable trends including age and sex interactions. A mayor finding was the combined influence of PA and dietary patterns on the LS7 scale as well as ponderal effects of lifestyle factors and MCS12/PCS12 among scores that suggest the need to implement policies and clinical interventions considering integrative factors in precision nutrition and PH actions.

## Data Availability

Data supporting the conclusions of this article will be available upon request from the study director, JAM.
